# Two subtle problems with overrepresentation analysis

**DOI:** 10.1093/bioadv/vbae159

**Published:** 2024-10-21

**Authors:** Mark Ziemann, Barry Schroeter, Anusuiya Bora

**Affiliations:** Bioinformatics Working Group, Burnet Institute, Melbourne, VIC 3004, Australia; School of Life and Environmental Sciences, Deakin University, Geelong, VIC 3216, Australia; School of Life and Environmental Sciences, Deakin University, Geelong, VIC 3216, Australia; Bioinformatics Working Group, Burnet Institute, Melbourne, VIC 3004, Australia; School of Life and Environmental Sciences, Deakin University, Geelong, VIC 3216, Australia

## Abstract

**Motivation:**

Overrepresentation analysis (ORA) is used widely to assess the enrichment of functional categories in a gene list compared to a background list. ORA is therefore a critical method in the interpretation of ’omics data, relating gene lists to biological functions and themes. Although ORA is hugely popular, we and others have noticed two potentially undesired behaviours of some ORA tools. The first one we call the ‘background problem’, because it involves the software eliminating large numbers of genes from the background list if they are not annotated as belonging to any category. The second one we call the ‘false discovery rate problem’, because some tools underestimate the true number of parallel tests conducted.

**Results:**

Here, we demonstrate the impact of these issues on several real RNA-seq datasets and use simulated RNA-seq data to quantify the impact of these problems. We show that the severity of these problems depends on the gene set library, the number of genes in the list, and the degree of noise in the dataset. These problems can be mitigated by changing packages/websites for ORA or by changing to another approach such as functional class scoring.

**Availability and implementation:**

An R/Shiny tool has been provided at https://oratool.ziemann-lab.net/ and the supporting materials are available from Zenodo (https://zenodo.org/records/13823301).

## 1 Introduction

Overrepresentation analysis (ORA) is a type of functional enrichment analysis (FEA) that involves the summarization of omics data to reflect biological differences. FEA has become one of the most popular methods in bioinformatics, collectively accumulating 131 332 citations as of late 2019 (Xie *et al.* 2021).

ORA involves the selection of genes of interest, followed by a test to ascertain whether certain functional categories are overrepresented in the selected genes. Since its initial development in 1999 (Tavazoie *et al.* 1999), ORA has proliferated widely, becoming part of many bioinformatics websites and software packages. The most popular ORA website is DAVID (Dennis *et al.* 2003, Huang *et al.* 2009, Sherman *et al.* 2022), followed by Ingenuity Pathway Analysis, a commercial software package provided by QIAGEN Inc. ORA has been implemented into R/Bioconductor packages including clusterProfiler (Yu *et al.* 2012, Wu *et al.* 2021), limma (Ritchie *et al.* 2015), and GOseq (Young *et al.* 2010).

Another popular approach to FEA is functional class scoring (FCS). In FCS, all detected genes are ranked by their degree of differential expression followed by a statistical test for enrichment of gene categories at either extreme of the ranked list (Subramanian *et al.* 2005).

According to a study that used simulated differential expression data, FCS accuracy was superior to ORA over a range of experimental designs (Kaspi and Ziemann 2020). A study of 1366 PubMed Central articles featuring enrichment analysis published in 2019 indicates that seven of the top eight most popular tools were based on ORA (according to the Supplementary Data) (Wijesooriya *et al.* 2022). The discrepancy between performance and popularity is likely due to the relative ease of conducting ORA as compared to FCS. A minimal ORA involves pasting a list of gene symbols into a text box on a website, with results appearing nearly instantly. On the other hand, FCS typically involves installing specific software and dealing with a dataset representing every detected gene (∼20 000 rows) and ensuring that the file format from upstream tools is compatible with FCS packages.

Despite the popularity of ORA, there are concerns that this technique is being misused. Due to technological and biological reasons, not all genes are detected in transcriptomic studies, meaning that some genes will more readily appear as differentially expressed. To address this bias, a background list of detected genes is required for comparison to the list of selected genes (foreground list) (Tilford and Siemers 2009). Failure to use a background gene list leads to dramatic changes in enrichment results, rendering them unreliable (Timmons *et al.* 2015, Wijesooriya *et al.* 2022). Unfortunately, use of an appropriate background list is reported in only a small fraction (∼4%) of peer-reviewed articles describing enrichment analysis (Wijesooriya *et al.* 2022). Correction of *P*-values for multiple testing is also crucial in controlling the false positive rate, as enrichment analysis typically involves thousands of parallel tests (Tilford and Siemers 2009). But appropriate *P*-value correction is reported in only ∼54% of studies (Wijesooriya *et al.* 2022). These issues have resulted in the development of best practices for end users and stronger reporting standards (Zhao and Rhee 2023).

The focus of this work is to raise awareness of two existing problems in ORA implementations of some popular enrichment tools. The first problem is that genes belonging to the background list are removed from the analysis entirely if they are not annotated as belonging to any functional categories. This results in the background list appearing smaller than it should, leading to an underestimation of fold enrichment scores and significance values. In principle, this problem would differentially affect analyses involving different gene set libraries, with smaller libraries such as Kyoto Encyclopedia of Genes and Genomes (KEGG) (Kanehisa *et al.* 2023), which describes 2727 genes affected more severely as compared to larger libraries like Gene Ontology (GO) (Gene Ontology Consortium *et al.* 2023), which describes 19 428 genes, figures obtained from MSigDB June 2024 (Liberzon *et al.* 2015).

The second problem is that adjusting *P*-values for multiple testing, also known as false-discovery rate correction, is sometimes implemented improperly. Determining which pathways should be subject to false-discovery rate correction depends on how a detection threshold is set. Ideally, a pathway should be considered detected based on the presence of a predetermined number of member genes in the background set. However, some tools use the foreground set to define a pathway as detected. This is problematic, as the act of trying to find an intersection between a foreground gene list and a pathway is a test, even if there are no common genes. This results in pathways being discarded from the analysis if no common genes are found in the foreground, despite many genes being present in the background list. This artificially reduces the number of tests conducted and makes FDR values appear lower than they should, potentially raising the rate of false positives. We expect this problem to have a more severe effect when the foreground list is small.

Here, we define the effect of these two problems on real RNA-seq-based enrichment analysis results and demonstrate how these two issues impact performance using simulated expression data. Finally, we classify popular tools regarding these two problems and provide general recommendations for end users.

## 2 Methods

### 2.1 Expression data preparation

To quantify the effect of these two problems on RNA-seq-based enrichment analysis, data from seven transcriptomic studies were downloaded from DEE2 using the getDEE2 R/Bioconductor package (Ziemann *et al.* 2019). The criteria for selection included humans as the species, presence in the DEE2 database, passing most DEE2 quality control measures, a simple control-case experiment design with two or three replicates per group, and with 50 or more differentially expressed genes (FDR < 0.05). Raw data for these seven studies are available from NCBI Sequence Read Archive under the accession numbers SRP128998, SRP038101, SRP037718, SRP096177, SRP097759, SRP253951, and SRP068733 (Keating *et al.* 2014, Lund *et al.* 2014, Rafehi *et al.* 2014, 2017, Blanco-Melo *et al.* 2020, Felisbino *et al.* 2021). For each dataset, kallisto (v0.43.1) (Bray *et al.* 2016) transcript counts were aggregated to the gene level. Genes with fewer than 10 reads per sample on average were removed from downstream analysis. The remaining genes passing this selection were included in the background gene set. Differential expression analysis was conducted with DESeq2 v1.44.0 (Love *et al.* 2014), with genes identified by their Ensembl identifiers. Gene symbols were then fetched using biomaRt v2.60.0, based on Ensembl version 112 (Durinck *et al.* 2009). Descriptive information of the seven datasets is provided in Table 1.

**Table 1.** Seven selected RNA-seq datasets for benchmarking.

SRA accession	Control accessions	Case accessions	Comparison	No. detected genes	No. genes with FDR < 0.05	Reference
SRP128998	SRR6467485, SRR6467486, SRR6467487	SRR6467479, SRR6467480, SRR6467481	Normal glucose versus high glucose	15 635	3472	(Felisbino *et al.* 2021)
SRP038101	SRR1171523, SRR1171524, SRR1171525	SRR1171526, SRR1171527, SRR1171528	Control versus azacytidine treated cells	13 926	3590	(Lund *et al.* 2014)
SRP037718	SRR1168228, SRR1168229, SRR1168230	SRR1168225, SRR1168226, SRR1168227	Control versus SAHA treated cells	15 477	4988	(Rafehi *et al.* 2014)
SRP096177	SRR5150595, SRR5150596, SRR5150597	SRR5150592, SRR5150593, SRR5150594	Control versus Set7 knock-down cells	15 607	5152	(Keating *et al.* 2014)
SRP097759	SRR5201525, SRR5201526	SRR5201527, SRR5201528	GFP control versus SAHH overexpression	19 139	62	Unpublished
SRP253951	SRR11517674, SRR11517675, SRR11517676	SRR11517677, SRR11517678, SRR11517679	Mock versus SARS-CoV-2 infection	15 182	8588	(Blanco-Melo *et al.* 2020)
SRP068733	SRR3112216, SRR3112217, SRR3112218	SRR3112219, SRR3112220, SRR3112221	Control versus EP300 knock-down	14 255	7365	(Rafehi *et al.* 2017)

### 2.2 Examining the background problem using real transcriptome data

Human gene sets were obtained from MSigDB v2023.2 (Liberzon *et al.* 2015), and included KEGG Medicus (Kanehisa *et al.* 2023), Reactome (Milacic *et al.* 2024), Wikipathways (Agrawal *et al.* 2024), MicroRNA targets from miRdb (Chen and Wang 2020), transcription factor targets from GTRD (Kolmykov *et al.* 2021), GO (Gene Ontology Consortium *et al.* 2023), Human Phenotype Ontology (HPO) (Köhler *et al.* 2021) and Cell markers and Hallmark pathways (Liberzon *et al.* 2015). To demonstrate the background problem, differentially expressed genes with FDR < 0.05 were subset, with separate lists for up- and down-regulated genes. If fewer than 200 genes met this criterion, then the 200 genes with the smallest *P*-values were selected, as suggested by a previous report (Tarca *et al.* 2013). These gene lists were subjected to ORA using clusterProfiler’s enricher function v4.12.0, using a minimum set size of 5 and no maximum set size.

To systematically assess the impact of the background problem, we conducted ORA with clusterProfiler using the different gene set libraries described above for the seven RNA-seq datasets. ClusterProfiler was used with the same parameters as above, and we devised a simple workaround to the background problem, which is to append the entire background list as a gene set to the library. This forces clusterProfiler to retain all background genes. The ORA results were then filtered for significant sets using an FDR threshold of 0.05. Jaccard index was used to compare the original and corrected analyses.

### 2.3 Examining the FDR problem

For this test, 2000 of the top up- and down-regulated genes were selected from each differential expression dataset based on *P*-value. A workaround for the FDR problem was devised by filtering the gene sets by the presence of at least two genes in the background list prior to running clusterProfiler. After running clusterProfiler using the parameters above, the number of gene sets in the results was quantified, and compared to the number that should have been reported using the two gene limit. The difference between these numbers (*n*) represents the number of missing gene sets. To account for these tests, *n* values of 1 were appended to the *P*-values obtained by clusterProfiler, followed by FDR correction in R, to obtain the properly corrected FDR values. As above, the analysis of seven datasets was done with nine gene set libraries, the significant sets were selected and the Jaccard statistics were collected for each run to compare original and corrected analysis.

As we predicted that this effect could be more severe for smaller foreground lists, we performed parallel analysis with foreground gene lists with sizes varying between 125 and 2000 genes, as ranked by FDR value.

### 2.4 Quantifying the effect of these problems on precision and recall using simulated expression data

RNA-seq counts for SRA run accession ERR2539161 were obtained from DEE2 (Mo *et al.* 2018). This dataset was selected due to its high number of assigned reads (367M). A library of 200 random gene sets, each containing 30 genes, was generated. To generate differential expression profiles, 5% of gene sets were selected to be differentially expressed, with equal numbers of up- and down-regulated pathways. Pseudosamples were generated by first down-sampling the counts using the thincounts function of the edgeR package v4.2.1 (Robinson *et al.* 2010) to 20M reads, followed by the addition of some extra variation drawn from a normal distribution with mean of 1 and SD varying between 0 and 0.6, this value selected randomly for each gene. Three ‘control’ and ‘case’ pseudosamples were generated. For the genes selected to be differentially expressed, expression values were multiplied by a log-fold change in the case samples of 0.5 for up-regulated genes and −0.5 for down-regulated genes. Genes that were selected to be both up- and down-regulated were left unchanged. With these expression profiles, DESeq2 was used for differential analysis, and the results were subjected to clusterProfiler which suffers from both problems, clusterProfiler with the background problem fix, clusterProfiler with the FDR problem fix, clusterProfiler with the fixes to background and FDR problems, an ORA function called fora from fgsea bioconductor package v1.30.0 that does not suffer these problems, and fgsea a fast implementation of the preranked gene set enrichment analysis (GSEA) algorithm (Korotkevich *et al.* 2016). For the ORA functions above, significantly differentially expressed genes with FDR < 0.05 were selected for the foreground gene set. If fewer than 200 genes were detected as significant, then 200 genes with the smallest *P*-values were used for ORA, like a previous report (Tarca *et al.* 2013). For fgsea, the DESeq2 test statistic was used for scoring differential expression. After enrichment analysis with these approaches, the significant up- and down-regulated sets were selected and compared to the ground truth to calculate precision, recall, and F1 score. At each value of added variance, 1000 replications were conducted. No set seed was used, but results were similar when run on different computer systems. All analyses were undertaken with R v4.4.1 in a Docker container.

### 2.5 Comparing ORA and FCS methods by down-sampling real RNA-seq data

To understand the sensitivity and false positive rate of ORA and FCS methods, we employed a down-sampling approach to a previously published dataset derived from 37 lung cancer patients, having tumour and normal tissues for each patient (Cho *et al.* 2022). These RNA-seq-based gene expression counts were downloaded from NCBI GEO under accession number GSE158420. After reading them into R, we noticed some gene names were converted to dates (Abeysooriya *et al.* 2021), so we used the HGNChelper package to fix them (Oh *et al.* 2020). DESeq2 was then used for differential expression analysis correcting for patient-of-origin to identify genes differentially expressed in tumour compared to normal tissue. FCS was conducted using fgsea using the DESeq2 test statistic to score each gene. ORA was conducted using fora, as above. For the ORA background, genes with a mean of 10 or more reads per sample on average across the comparison were included. Reactome pathways were used. For down-sampling, sample size was varied between 2 and 30 patients, selected pseudorandomly with a set seed, followed by parallel analysis with fgsea and fora pipelines, using a significance filter of FDR < 0.05. We then calculated the number of pathways identified in down-sampled data that were consistent with the full dataset. The number of consistent pathways gives an indication of the sensitivity of each method. Then, we calculated the number of pathways identified in the down-sampled data that were inconsistent with the full dataset. The proportion of significant pathways classified as inconsistent provides an estimate of the real false discovery rate. This was repeated 50 times at each sample size, and the results and the results were presented as a violin plot.

## 3 Results

### 3.1 Understanding the background problem

**Figure 1.**
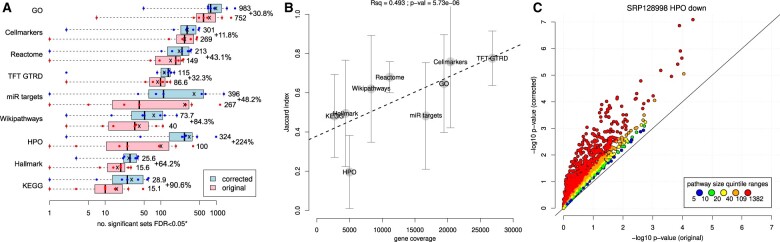
Characterization of the background problem. (A) Number of statistically significant pathways (FDR < 0.05) in seven independent RNA-seq studies with and without correction of the background problem. Separate tests were conducted for up- and down-regulated gene lists. Mean values are depicted as an ‘X’. The percentage increase in the mean number of significant sets is shown. ‘*’ indicates that 1 has been added to all values before log transformation. (B) Impact of the background problem is worse for smaller gene set libraries. Mean Jaccard similarity index was calculated for original and corrected ORA for seven independent datasets. The number of genes represented in one or more functional sets is shown on the *x*-axis, and the mean Jaccard index is shown on the *y*-axis; whiskers represent the SD. (C) Significance values (−log10 *P*-values) for dataset #1 (SRP128998) enrichment analysis using HPO gene sets with and without correcting the background problem. Gene sets were divided into quintiles based on the number of genes detected in the dataset.

A workaround to eliminate the background problem was developed and we used it to compare the results of ORA of seven datasets with the original and corrected methods for nine different gene set libraries. The number of statistically significant results was calculated for each of the gene set libraries before and after correcting the problem (Fig. 1A). Each study was affected to a similar degree (31%–94% increase), except for #5 which saw the number of significant GO terms increase from 8 to 54 after correction (Supplementary Table S1). The similarity between results, as quantified with the Jaccard similarity, was highest for Cellmarkers and GTRD at 0.76 and 0.77, respectively, and the lowest similarity was recorded for HPO and KEGG with 0.20 and 0.48, respectively. Observed mean Jaccard scores correlated with the number of genes annotated to one or more functional categories in the gene set library (Fig. 1B). This result suggests that ORA with smaller gene set libraries like HPO and KEGG are impacted more severely. We investigated discrepant results for dataset #1 (SRP128998) down-regulated genes in HPO gene sets. A scatterplot of significance scores (Fig. 1C) shows severe deviation in *P*-values in gene sets in the largest quintile (109–1382 members detected), suggesting larger gene sets are more severely affected by the background problem.

### 3.2 Understanding the FDR problem

**Figure 2.**
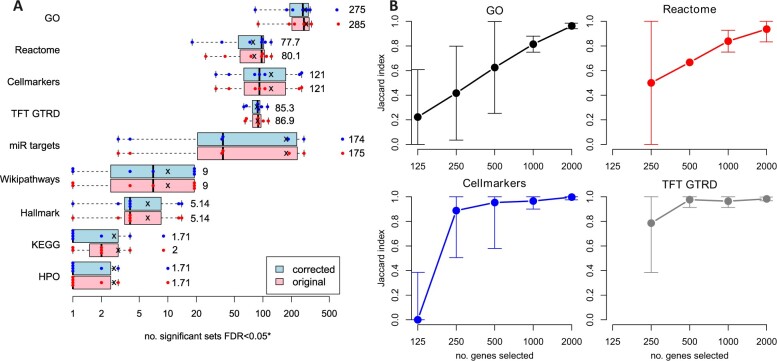
Characterization of the FDR problem. (A) Number of statistically significant pathways (FDR < 0.05) in seven independent RNA-seq studies with and without the FDR problem. The foreground list consisted of 2000 genes with the smallest *P*-values. Mean values are depicted as an ‘X’. ‘*’ indicates that 1 has been added to all values before log transformation. (B) Impact of the FDR problem is worse for shorter foreground gene lists. The mean Jaccard index between original and corrected analysis was calculated for seven RNA-seq datasets with gene sets from GO, Reactome, Cellmarkers, and TFT GTRD; whiskers represent the SD.

A workaround for the FDR problem was developed, then original and corrected analyses were conducted for the seven datasets and nine gene set libraries. Results showed no major differences in the number of significant sets between original and corrected analyses (Fig. 2A). The impact of the FDR problem was similar across these seven datasets (Supplementary Table S2).

We posited that a shorter foreground gene list length might exacerbate the problem, so we performed parallel analysis with foreground lists of length between 125 and 2000 genes (Fig. 2B). A clear trend was observed for GO, Reactome, Cellmarkers, and TFT GTRD, with Jaccard similarity being significantly reduced when foreground gene lists were shorter than 500 genes. Interestingly, the drop in Jaccard similarity was more severe for GO and Reactome sets as compared to Cellmarkers and TFT GTRD sets and may be associated with the size of the gene sets in the library. Median gene set sizes for these are GO:18, Reactome:11, TFT GTRD:287, and Cellmarkers:115. These results demonstrate that the FDR problem is more severe when dealing with shorter foreground lists, and this is exacerbated when functional categories in the gene set library are also small.

### 3.3 Impact of these two problems on accuracy determined with simulated data

**Figure 3.**
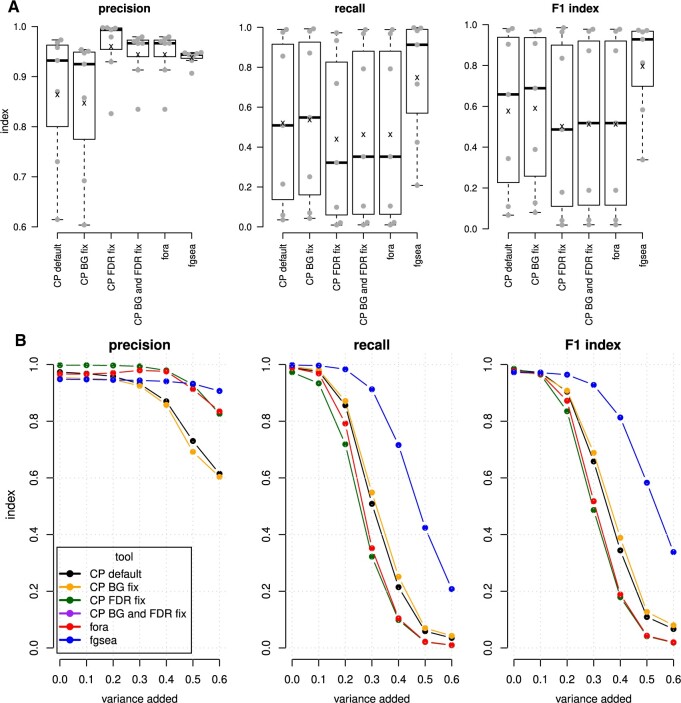
Impact of correcting these two problems on the accuracy of simulated expression data. (A) Precision, recall, and F1 scores across simulations. CP; clusterProfiler, CP BG fix; clusterProfiler with a workaround to the background problem, CP FDR fix; clusterProfiler with a workaround to the FDR problem, CD BG, and FDR fix; clusterProfiler with mitigations for both background and FDR problems, fora; ORA function of the fgsea package, fgsea; an FCS method similar to GSEA. Mean values are depicted as an ‘x’. (B) Precision, recall, and F1 score are shown with the addition of different amounts of random noise to expression levels. The line for clusterProfiler with mitigations for both problems is not visible as the performance was identical to fora.

Simulated differential expression profiles with *a priori* changes to predetermined pathway genes were analysed in parallel by clusterProfiler default and with mitigations for problems described above. In addition, these data underwent ORA with fora which does not suffer from these two problems. For comparison, fgsea was used to compare the performance of these two ORA methods against a prototypical FCS method. These methods were tested with different levels of introduced variation and the precision, recall, and F1 scores were recorded (Fig. 3A). Mitigating the background problem improved mean recall (by 2.9%), however, it also caused a decrease in mean precision (1.3% lower). Addressing the FDR problem improved mean precision by 11.7%, but mean recall dropped by 16.6%. Addressing both problems improved mean precision by 9.7% but decreased mean recall by 11.7%. Under these conditions, the FDR problem affected results more severely than the background problem. Results obtained for clusterProfiler after implementing the two mitigations were identical to fora to three significant figures. fgsea recorded mean precision of 0.939, similar to the ORA methods, but recall was far superior, with fgsea scoring 44.9% higher than default clusterProfiler and 64.2% higher than fora. As a result, fgsea’s overall accuracy as defined by F1 index was 35.4% higher than fora and 28.9% higher than default clusterProfiler.

When looking at the accuracy profile at different levels of added noise (Fig. 3B, Supplementary Fig. S1), ORA techniques show high precision when the added noise is low (<0.2), but precision drops at higher levels of added noise. The rate of drop in precision was more severe for approaches that suffer from the FDR problem. The precision of fgsea was relatively stable (ranging from 0.95 to 0.91) despite high levels of added noise. The recall of fgsea was superior to all ORA approaches across all levels of added noise.

### 3.4 Exploring sensitivity in real RNA-seq data

**Figure 4.**
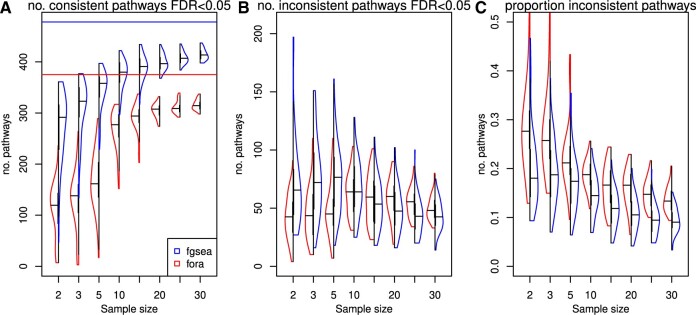
Down-sampling confirms higher sensitivity for FCS over ORA. (A) Violin plot showing the number of significant pathways identified in down-sampled data that are consistent with findings in the full dataset, using FCS (fgsea) and ORA (fora) methods. Down-sampling was repeated 50 times. The horizontal lines indicate the number of significant pathways identified in the full set of 37 patients using fgsea (upper, blue) and fora (lower, red). (B) The number of significant pathways identified in down-sampled data that are not consistent with the full dataset. (C) The proportion of pathways in down-sampled data that are not consistent with the full dataset.

To understand whether FCS has better sensitivity than ORA in a real setting, we conducted a systematic down-sampling of a gene expression dataset consisting of normal and tumour samples of 37 lung cancer patients. In the full set of patient samples, fora (ORA) yielded 375 significantly differentially regulated Reactomes, while fgsea (FCS) gave 480 (FDR < 0.05). Fgsea yielded consistently more significant pathways than fora in down-sampled data (Fig. 4A). Fgsea also identified a larger proportion of pathways in down-sampled data compared to fora. For example, at a sample size of 5, fgsea identified 73.3% (median) of pathways from the full set as compared to 41.5% for fora, confirming better recall for FCS over ORA. It was noted that fgsea identified more pathways in down-sampled data that were inconsistent with the full dataset as compared to fora (Fig. 4B). However, inconsistent pathways were a smaller proportion of all findings for fgsea as compared to fora (Fig. 4C), indicating an overall lower false positive rate for FCS compared to ORA methods.

## 4 Discussion

The background problem came to our attention when a doctoral student in our department was puzzled by results obtained from clusterProfiler when using a custom set of pathways. This exact issue was the subject of several posts to online bioinformatics forums dating to ∼2020 where confused users were wondering why the number of background genes changed so much. The effect of excluding genes without functional annotations causes the background list to appear smaller than it would otherwise be, causing enrichment scores to appear smaller. The results shown here indicate the background problem affects smaller gene set libraries like KEGG and HPO more severely as compared to Cellmarkers and GO libraries which describe a larger proportion of all genes. ClusterProfiler maintainers recently provided an option to prevent the loss of unannotated genes from the background options(enrichment_force_universe=TRUE), however, this feature is not yet described in the official documentation.

The FDR problem is potentially more serious in that it could lead to increasing false positives under specific conditions; when the foreground list is short, and the median pathway size is relatively small. These false positives arise due to an underestimate of the actual number of tests conducted. In extreme cases, correction of this problem can lead to around half of the statistically significant results changing (see Fig. 2B).

So end-users can explore the effect of these two problems on their work, we have developed an R/Shiny web application (see ‘Availability of Data and Materials’ below). Users can upload their foreground and background lists, select their preferred gene set library and compare results from default and patched analyses.

ClusterProfiler is not the only tool to suffer from these problems, they are widespread (Table 2). Still, there are some tools free of such problems. For example, fora is a good option for R-based workflows, however, the function does not calculate the fold enrichment score, so users will need to do it themselves (Korotkevich *et al.* 2016). ShinyGO is an excellent alternative for web-based analysis (Ge *et al.* 2020). It does not suffer from the two issues described here; it is easy to use and has a high degree of reproducibility thanks to its stand-alone versioned docker container option for local execution. One problem that all of these tools listed in Table 2 have is that a background list is optional. Sometimes this feature is hidden under ‘advanced options’. Given the strong biological and technical biases that are present in contemporary omics such as single cell and spatial transcriptomics, it should be a mandatory step for users to provide a custom background list.

**Table 2.** Classification of several popular freely available ORA tools regarding the provision of information such as enrichment scores and FDR values, as well as behaviours regarding the two algorithmic issues described above.

Tool	Version	Type	Provides FDR values	Provides enrichment score	Proper background handling	Proper FDR	Reference
DAVID	v2023q4	Web	Yes	Yes	No	Yes	(Sherman *et al.* 2022)
Panther	v19	Web	Yes	Yes	Yes	Yes	(Mi *et al.* 2019)
Enrichr	June 2023	Web	Yes	Yes	Yes	No	(Kuleshov *et al.* 2016)
KOBAS-i	KOBAS 3.0	Web	Yes	No	Yes	No	(Bu *et al.* 2021)
WebGestalt	2024	Web	Yes	Yes	No	Yes	(Elizarraras *et al.* 2024)
g: Profiler	Feb 2024	Web	Yes	No	No	Yes	(Kolberg et al. 2023)
STRING-DB	v12.0	Web	Yes	Yes	Yes	Yes	(Szklarczyk *et al.* 2023)
ShinyGO	v0.80	Web	Yes	Yes	Yes	Yes	(Ge *et al.* 2020)
BinGO	v3.0.5	Cytoscape	Yes	No	No	No	(Maere *et al.* 2005)
ClueGO	v2.5.10	Cytoscape	Yes	No	No	No	(Bindea et al. 2009)
clusterProfiler	v4.12.0	R package	Yes	No	No	No	(Wu *et al.* 2021)
topGO	v2.56.0	R package	No	No	No	Yes^a^	(Alexa and Rahnenfuhrer 2024)
Goseq	v1.56.0	R package	No	No	No^b^	Yes^a^	(Young *et al.* 2010)
goana/kegga	limma v3.60.3	R package	No	No	No^c^	Yes^a^	(Ritchie *et al.* 2015)
for a	fgsea v1.30.0	R package	Yes	No^d^	Yes	Yes	(Korotkevich et al. 2016)

a Denotes that although no FDR values are provided, the results include sets where no overlaps were found, so users can adjust raw *P*-values themselves.

b Denotes functions where options are provided to adjust this behaviour and are documented in the user manual.

c Documentation states unannotated genes are discarded with default settings using whole genome background, but not when a custom background is used.

d Denotes enrichment scores will be included in the next Bioconductor release.

The purpose of this work was to thoroughly characterize two subtle problems with ORA, but there are some bigger problems noted. Firstly, all variations of ORA were less accurate (based on F1 index) as compared to FCS in this simulation work (Fig. 3A), confirming a previous report (Kaspi and Ziemann 2020). Specifically, the precision of ORA deteriorated with greater levels of added noise, while FCS was relatively stable. This means researchers can be more confident with FCS results, even if the data are noisy. The higher recall of FCS demonstrated in simulated differential expression profiles and confirmed with down-sampled real cancer RNA-seq data means that researchers will have more power to identify pathways relevant to the biological processes being studied. Secondly, many popular ORA tools listed in Table 2 do not provide enrichment scores in their default output tables. Enrichment scores are a surrogate for effect size, and without them, users may over-rely on significance values when interpreting enrichment results. Lastly, ORA results can vary dramatically depending on the number of genes selected in the foreground, a mostly arbitrary choice.

These issues together with previously mentioned pitfalls such as lack of background correction and methodological misreporting (Timmons *et al.* 2015, Wijesooriya *et al.* 2022), suggest that if the data can be scored/ranked, methods like FCS are strongly recommended over ORA. ORA is better suited to other cases where genes are classified into binary groups; for example, when investigating the overrepresented categories in gene expression modules (Langfelder and Horvath 2008), or with variant-harbouring genes associated with a disease (White *et al.* 2019). In these cases, we would recommend using a tool from Table 2 that reports FDR values and enrichment scores, and avoids background and FDR problems. Doubts also arise when choosing options and parameters for ORA. The most important is to use a background gene list that reflects the true universe of genes detected robustly in the assay. Secondly, the significance of enrichment results must be based on the FDR-corrected *P*-values. Selection criteria for foreground genes is another crucial choice; for this, we endorse the approach of Tarca *et al.*, (2013) which accommodates cases where relatively few individual genes meet the FDR threshold. Some ORA tools have a filter for gene set size. We would recommend a minimum size of 5–10 in order to avoid missing small gene sets that exhibit strong enrichments. Interpret with caution enrichment results where the intersection of foreground gene list and pathway is ≤3 genes. A maximum gene set filter is not required if end results are interpreted using enrichment scores together with FDR values; i.e. a small FDR value may be biologically uninteresting if the enrichment score is small. The choice of pathway database also influences the conclusions that can be drawn (Mubeen *et al.* 2020, Karp *et al.* 2021). Our suggestion is to use comprehensive and open-source pathway databases such as Reactome and Gene Ontology Biological Process. Proprietary databases like KEGG and Ingenuity are less comprehensive, have restrictive conditions for nonacademic users and do not provide public access to historical database versions which inhibits future reproducibility.

## Supplementary Material

vbae159_Supplementary_Data

## Data Availability

Publicly available data were obtained from Digital Expression Explorer 2 (dee2.io) and NCBI GEO using the accession numbers mentioned in the Methods section. Reproduction of the results here can be achieved using the GitHub code repository GitHub (https://github.com/markziemann/background) and Docker image (https://hub.docker.com/r/mziemann/background), which have both been deposited to Zenodo for long-term preservation (https://zenodo.org/record/13823301). The R/Shiny tool for comparing ORA with and without the two problems is currently available at https://oratool.ziemann-lab.net/ and available as a downloadable docker container for local execution (https://hub.docker.com/repository/docker/mziemann/background_app/general).
