# Supplementary material for: Two subtle problems with overrepresentation analysis
Source: Bioinform Adv. 2024 Oct 21;4(1):vbae159. doi: 10.1093/bioadv/vbae159 (PMC11557902; doi:10.1093/bioadv/vbae159)
Supplement: vbae159_Supplementary_Data [file vbae159_supplementary_data.zip › Table S2.docx]

Table S2. Number of statistically significant gene sets across nine gene set libraries and seven datasets using ORA with and without the FDR problem.

| **FDR problem** | **d1** | **d2** | **d3** | **d4** | **d5** | **d6** | **d7** |
| --- | --- | --- | --- | --- | --- | --- | --- |
| Cellmarkers original | 82 | 255 | 91 | 31 | 43 | 104 | 240 |
| Cellmarkers corrected | 81 | 255 | 91 | 31 | 43 | 104 | 242 |
| GO original | 289 | 621 | 269 | 88 | 214 | 188 | 323 |
| GO corrected | 289 | 600 | 262 | 82 | 205 | 177 | 313 |
| Hallmark original | 3 | 13 | 3 | 2 | 0 | 12 | 3 |
| Hallmark corrected | 3 | 13 | 3 | 2 | 0 | 12 | 3 |
| HPO original | 9 | 2 | 1 | 0 | 0 | 0 | 0 |
| HPO corrected | 9 | 2 | 1 | 0 | 0 | 0 | 0 |
| KEGG original | 3 | 1 | 1 | 8 | 0 | 1 | 0 |
| KEGG corrected | 3 | 0 | 0 | 8 | 0 | 1 | 0 |
| miR targets original | 188 | 37 | 3 | 2 | 270 | 690 | 36 |
| miR targets corrected | 184 | 37 | 3 | 2 | 269 | 688 | 36 |
| Reactome original | 122 | 96 | 98 | 108 | 39 | 74 | 24 |
| Reactome corrected | 121 | 96 | 97 | 105 | 36 | 72 | 17 |
| TFT GTRD original | 88 | 88 | 100 | 66 | 113 | 89 | 64 |
| TFT GTRD corrected | 88 | 87 | 96 | 66 | 111 | 87 | 62 |
| Wikipathways original | 18 | 18 | 3 | 0 | 0 | 18 | 6 |
| Wikipathways corrected | 18 | 18 | 3 | 0 | 0 | 18 | 6 |
| mean orig | 89.1 | 125.7 | 63.2 | 33.9 | 75.4 | 130.7 | 77.3 |
| mean corr | 88.4 | 123.1 | 61.8 | 32.9 | 73.8 | 128.8 | 75.4 |
| uplift | -0.7% | -2.0% | -2.3% | -3.0% | -2.2% | -1.4% | -2.4% |
