# Supplementary material for: Two subtle problems with overrepresentation analysis
Source: Bioinform Adv. 2024 Oct 21;4(1):vbae159. doi: 10.1093/bioadv/vbae159 (PMC11557902; doi:10.1093/bioadv/vbae159)
Supplement: vbae159_Supplementary_Data [file vbae159_supplementary_data.zip › Table S1.docx]

Table S1. Number of statistically significant gene sets across nine gene set libraries and seven datasets using ORA with and without the background problem.

| **Background problem** | **d1** | **d2** | **d3** | **d4** | **d5** | **d6** | **d7** |
| --- | --- | --- | --- | --- | --- | --- | --- |
| Cellmarkers original | 139 | 439 | 345 | 244 | 0 | 267 | 448 |
| Cellmarkers corrected | 170 | 477 | 382 | 286 | 1 | 297 | 491 |
| GO original | 485 | 1843 | 686 | 390 | 6 | 588 | 1263 |
| GO corrected | 707 | 2168 | 915 | 709 | 48 | 789 | 1544 |
| Hallmark original | 7 | 25 | 18 | 13 | 0 | 28 | 18 |
| Hallmark corrected | 20 | 38 | 21 | 27 | 2 | 37 | 34 |
| HPO original | 2 | 380 | 92 | 24 | 0 | 17 | 186 |
| HPO corrected | 42 | 988 | 241 | 283 | 1 | 265 | 449 |
| KEGG original | 9 | 25 | 2 | 14 | 0 | 47 | 9 |
| KEGG corrected | 19 | 59 | 6 | 24 | 0 | 56 | 38 |
| miR targets original | 4 | 40 | 21 | 281 | 0 | 1166 | 360 |
| miR targets corrected | 81 | 111 | 38 | 635 | 0 | 1365 | 544 |
| Reactome original | 64 | 239 | 94 | 207 | 0 | 254 | 185 |
| Reactome corrected | 102 | 351 | 159 | 307 | 0 | 334 | 240 |
| TFT GTRD original | 52 | 98 | 108 | 71 | 1 | 147 | 129 |
| TFT GTRD corrected | 85 | 140 | 135 | 115 | 1 | 173 | 153 |
| Wikipathways original | 33 | 108 | 8 | 13 | 1 | 82 | 35 |
| Wikipathways corrected | 46 | 193 | 12 | 50 | 1 | 97 | 117 |
| mean orig | 88.3 | 355.2 | 152.7 | 139.7 | 0.9 | 288.4 | 292.6 |
| mean corr | 141.3 | 502.8 | 212.1 | 270.7 | 6.0 | 379.2 | 401.1 |
| uplift | 60.0% | 41.5% | 38.9% | 93.8% | 575% | 31.5% | 37.1% |
