# Supplementary figures and images for: Two subtle problems with overrepresentation analysis
Source: Bioinform Adv. 2024 Oct 21;4(1):vbae159. doi: 10.1093/bioadv/vbae159 (PMC11557902; doi:10.1093/bioadv/vbae159)

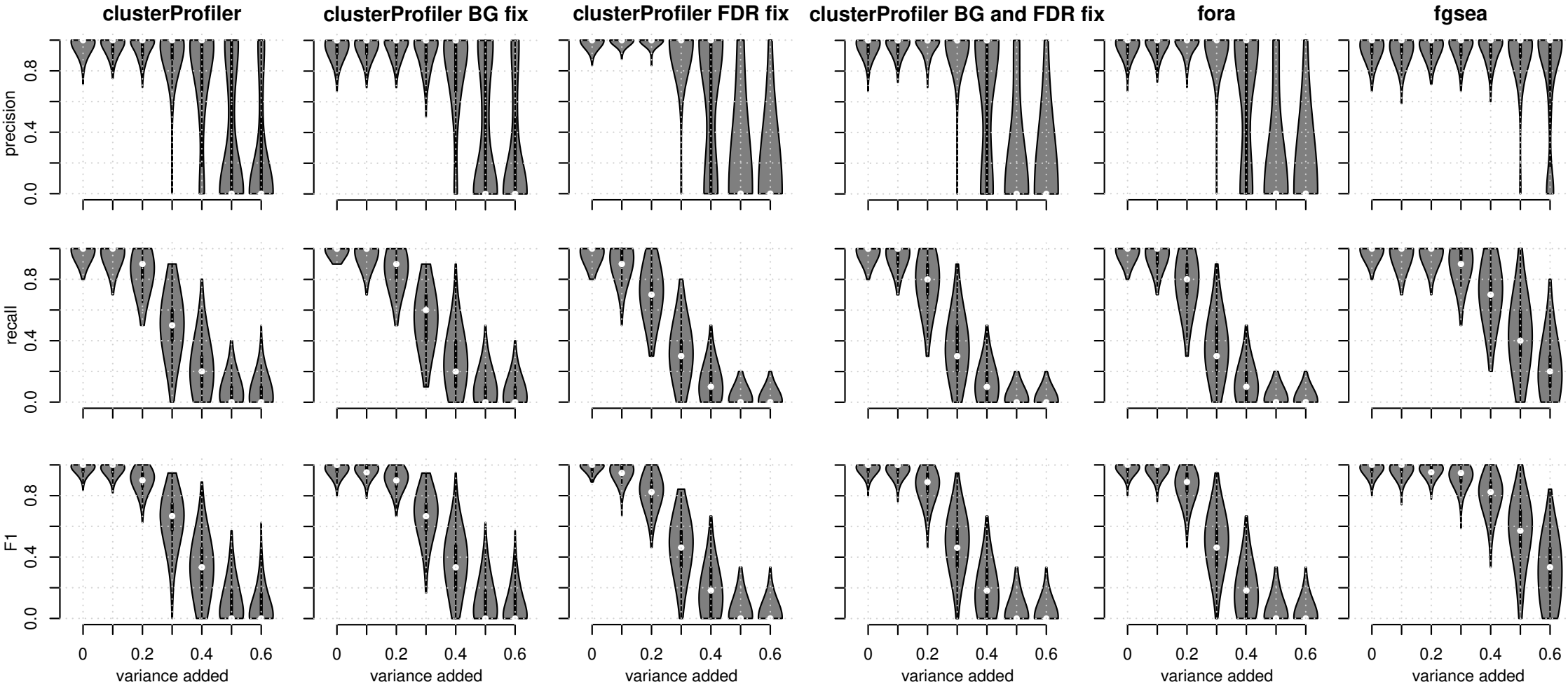

Supplement: vbae159_Supplementary_Data [file vbae159_supplementary_data.zip › FigS1.pdf]
